# Dry eye disease: identification and therapeutic strategies for primary care clinicians and clinical specialists

**DOI:** 10.1080/07853890.2022.2157477

**Published:** 2022-12-28

**Authors:** John Sheppard, Bridgitte Shen Lee, Laura M. Periman

**Affiliations:** a Virginia Eye Consultants, Eyecare Partners, Norfolk, VA, USA; b Vision Optique, Houston, TX, USA; c Periman Eye Institute, Seattle, WA, USA

**Keywords:** Dry eye disease, keratoconjunctivitis sicca, therapeutics, primary care, family medicine, specialty medicine

## Abstract

Dry eye disease (DED) is a multifactorial disorder characterized by loss of tear film homeostasis with an estimated worldwide prevalence of 5% to 50%. In DED, dysfunction of the ocular structures that create and regulate the tear film components—including the lacrimal glands, meibomian glands, cornea, and conjunctiva—causes a qualitative and/or quantitative tear deficiency with resultant tear film instability and hyperosmolarity. This initiates a vicious cycle of ocular surface inflammation and damage that may ultimately impair the quality of life and vision of affected patients. Many factors can contribute to the development of DED, including ocular and systemic diseases, topical and systemic medications, and environmental conditions. Because DED is a chronic disorder, treatment is most often long term and may utilize both pharmacologic and nonpharmacologic interventions to address all etiologic components. The long-term management of DED can be challenging and most often should involve eye care specialist referral. However, primary care clinicians (PCCs) are often the first points of contact for patients with DED and importantly provide initial diagnosis and preliminary patient education about the disease process. Consideration of DED is also vital for the practice of various specialties due to the large number of comorbidities and medications that can contribute to DED pathogenesis and progression. Therefore, it is important that PCCs and clinical specialists be aware of the etiology of DED and its available therapeutic options. This manuscript provides an overview of DED pathophysiology and treatment and discusses specific considerations regarding DED management for PCCs and clinical specialists.

**Key messages**
- Successful management of dry eye disease often requires the use of various pharmacologic and/or nonpharmacologic therapies, as well as environmental and lifestyle modifications, to mitigate the underlying etiologies and restore tear film homeostasis.
- Primary care clinicians play an essential role in dry eye disease management by establishing a diagnosis, educating patients about the disorder, and providing referrals to eye care specialists for initiation of specialized treatment and long-term follow-up.
- Primary care clinicians and clinical specialists should consider prescribing medications with fewer ocular surface effects whenever possible in patients at risk for or with existing dry eye disease.

## Introduction

Dry eye disease (DED) is a multifactorial disorder characterized by loss of tear film homeostasis that leads to a self-perpetuating cycle of ocular surface inflammation and damage [1,2]. Loss of tear film homeostasis is secondary to dysfunction of one or more ocular structures that create and regulate the tear film components, including the lacrimal glands, meibomian glands, cornea, and conjunctiva [1,3]. Many factors may affect these structures and contribute to the development of DED, including ocular disorders such as blepharitis and meibomian gland dysfunction, as well as various systemic diseases such as diabetes mellitus, Sjögren syndrome, rheumatoid arthritis, and systemic lupus erythematosus, among others [3]. Environmental factors such as low humidity, increased airflow over the ocular surface, dust, tobacco smoke, and air pollutants, as well as lifestyle influences like poor eyelid hygiene practices, extensive use of digital devices, and contact lens wear, can also promote DED [2,3]. In addition, several commonly prescribed topical and systemic medications may cause or exacerbate DED [2]. The resultant lack of normal tear production (aqueous deficient DED) and/or increase in tear evaporation (evaporative DED) leads to qualitative and/or quantitative tear deficiency, hyperosmolarity, and tear film instability [3,4]. This causes chronic ocular surface inflammation and injury that may ultimately impair vision in affected patients [3,5].

The estimated worldwide prevalence of DED ranges from 5% to 50% and varies by population [4,6]. Associated symptoms include ocular redness, dryness, itchiness, grittiness, foreign body sensation, eye fatigue, and visual disturbance [1,3,7]. Dry eye disease can adversely affect perceptions of both physical and mental wellbeing in affected patients, decreasing their overall quality of life [8,9]. In fact, studies have demonstrated that patients with moderate to severe DED rank the loss of utility associated with their disease similarly to patients affected by disorders such as disabling hip fracture and severe angina [10,11]. Dry eye disease has been found to negatively impact work productivity and activities of daily living, indicating significant socioeconomic impacts of the disorder [12,13].

Establishing a diagnosis of DED can be difficult, as signs and symptoms of the disease may not correlate with one another, and other conditions that may present similarly must be excluded [1,14]. Diagnosis is most often based on a combination of relevant patient history and multiple clinical diagnostics that detect tear film or ocular surface abnormalities [14]. These may include symptom questionnaires, ocular surface staining, lipid layer analysis, tear breakup time (TBUT), tear osmolarity, tear production, detection of ocular surface inflammatory markers, meibography, or eyelid health examination [14]. Once a diagnosis of DED has been made, treatment should be initiated as appropriate on an individualized basis.

In clinical practice, the diagnosis and management of DED can be challenging, and patients often require eye care specialist referral for long-term care [15,16]. However, DED is a common condition initially presented to primary care clinicians (PCCs) and is frequently associated with comorbidities treated by PCCs and clinical specialists [3,15]. This review aims to increase awareness of the etiology and pathophysiology of DED among PCCs and clinical specialists, inform these clinicians about the available pharmacologic and nonpharmacologic treatment options for DED, and discuss special considerations regarding DED management.

## Discussion

### Treatment of dry eye disease

The treatment of DED is often chronic and multifaceted to mitigate all etiologic components, and potentially involves both pharmacologic and nonpharmacologic interventions [2]. Treatment commonly progresses in a stepwise fashion, beginning with appropriate conservative therapies and advancing to more intensive treatments as indicated based on the severity of disease [2]. Overall goals of DED therapy are to restore and maintain ocular surface homeostasis, to minimize clinical signs and long-term ocular surface damage, and to ultimately maximize visual function and patient quality of life [2,4,16].

#### Pharmacological therapeutics

Many pharmacological agents are used in the treatment of DED, including topical and systemic medications. Commonly used topicals include lubricants, corticosteroids, lifitegrast, and cyclosporine A [2]. Systemic agents administered orally may include antibiotics (azithromycin and tetracyclines), poly-unsaturated omega-3 fatty acid supplements, and antioxidant supplements [2]. Additionally, varenicline nasal spray (Tyrvaya™, Oyster Point Pharma, Princeton, NJ) is a newly approved cholinergic agonist nasal spray indicated for the treatment of the signs and symptoms of DED [17,18]. Table 1 summarizes key information about the approved pharmacological agents available for DED treatment.

**Table 1.** Summary of available pharmacologic agents for the treatment of dry eye disease.

						
	Indication	Expected length of therapy	Mechanism of action	Contra-indications	Minimum age at treatment	Safety during pregnancy
**Topical agents**						
**Artificial tears**	Replace or supplement natural tear film in patients with DED [2]	Chronic [16]	Tear substitutes targeting 1 or more layers of the tear film [2]	Preserved solutions in patients requiring frequent dosing or using other chronic topical therapies [2]	Safe for pediatric use [19]	Refer to specific product information
**Corticosteroids**	Stop cycle of ocular surface inflammation inherent in DED [2]	Short term (several weeks) [2,16]	Anti-inflammatory and immunosuppressive effects mediated through multiple receptors [2]	Ocular hypertension, cataracts, active infection [2]	Use with caution in pediatric patients [19]	No adverse neonatal outcomes observed [20]
**Lifitegrast** (Xiidra^®^)	Treatment of the signs and symptoms of DED [21]	12 weeks [21]	Lymphocyte function–associated antigen-1 antagonist [21]	Hypersensitivity to lifitegrast or any other ingredients in formulation [21]	17 years [21]	No available data to inform drug-associated risk [21]
**Cyclosporine A**						
Restasis^®^	Increase tear production in patients whose tear production is presumed to be suppressed due to ocular inflammation associated with DED [22]	6 months [22]	Calcineurin inhibitor immunosuppressant [22]	Hypersensitivity to any ingredients in formulation [22]	16 years [22]	Maternal use not expected to cause fetal drug exposure [22]
Cequa™	Increase tear production in patients with DED [23]	12 weeks [23]	Calcineurin inhibitor immunosuppressant [23]	None [23]	18 years [23]	No adequately controlled studies to inform risk [23]
**Intranasal agents**						
**Varenicline** (Tyrvaya™)	Treatment of the signs and symptoms of DED [18]	4 weeks [18]	Nicotinic acetylcholine receptor agonist [18]	None [18]	Adult [18]	No available data to inform drug-associated risk [18]
**Systemic agents** **Antibiotics**						
Azithromycin	Treatment of blepharitis, MGD, and rosacea associated with DED [2]	5 days or pulse dosing for 1 month [2,24]	Stimulator of meibomian gland function with anti-inflammatory properties [2]	Hypersensitivity, use with antipsychotics or other drugs that prolong the QTc interval, use with P-glycoprotein substrates, long-term use in HSCT recipients [25]	Safe for pediatric use [26]	Considered generally safe for use during pregnancy [27,28]
Tetracyclines	Treatment of chronic anterior blepharitis, MGD, and rosacea associated with DED [2]	4–10 weeks or chronic low dose [2]	Broad-spectrum antibiotic with anti-inflammatory properties [2]	Hypersensitivity, pregnancy or breastfeeding, children under 16 years old, use with penicillin or isotretinoin [24,29,30]	16 years [29]	Use during pregnancy contraindicated [29,30]
**Supplements**						
Omega fatty acids	Inhibit proinflammatory cytokine production and T-lymphocyte proliferation implicated in DED [2]	At least 3 months [2]	Precursors of eicosanoids that modulate systemic inflammation [2]	Liver disease, atrial fibrillation, bleeding disorders [2]	Safe for pediatric use [31]	Only pharmaceutical-grade fish oil supplements safe for use during pregnancy [32]
Antioxidants	Prevent oxidative cellular injury that leads to ocular surface diseases like DED, improve ocular surface health [2,33]	At least 1 month [2,33]	Neutralize reactive oxygen species (free radicals) [33]	May increase risk of certain cancers, cardiovascular disease, or diabetes; use controversial in patients on chemotherapy [34]	Safe for pediatric use [35,36]	Limited studies of safety during pregnancy [37]

DED: dry eye disease; HSCT: hematopoietic stem cell transplant; MGD: meibomian gland dysfunction; QTc: corrected QT.

#### Nonpharmacological therapeutics

Nonpharmacological interventions used in the treatment of DED comprise procedural and device-based therapies as well as lifestyle modifications. Common procedural therapies include punctal occlusion, meibomian gland thermal pulsation and expression, intense pulsed light therapy, low-level light therapy, and microblepharoexfoliation [2,38]. Device-based therapies include eyelid hygiene devices, neurostimulation devices, heat or moisture goggles, and warm compresses [2,39]. Table 2 summarizes key information regarding these procedural and device-based therapies.

**Table 2.** Summary of available nonpharmacologic therapeutics for the treatment of dry eye disease.

	Indication	FDA approval	Expected length of therapy	Mechanism of action	Contraindications	Minimum age at treatment
**Procedural therapies**						
**Punctal occlusion**	Treatment of disorders benefitting from tear retention on the ocular surface, such as DED [2]	Yes [40,41]	Short term (absorbable punctal plugs), long term (nonabsorbable punctal plugs), or permanent (surgical occlusion) [2]	Physically blocks tear drainage with punctal plugs or surgical punctal occlusion [2]	Hypersensitivity to plug material, lacrimal pathway obstruction, ectropion, active ocular infection [42]	Use with caution in pediatric patients [19,43]
**Meibomian gland thermal pulsation** (Lipiflow^®^)	Application of localized heat and pressure in adults with chronic cystic eyelid disorders including MGD (evaporative DED) [44,45]	Yes [45]	Every 3–12 months as needed [44]	Applies heat to both inner eyelids to liquefy contents while pulsating pressure is simultaneously applied to outer eyelids to evacuate meibomian glands [44]	Ocular surgery or injury within 3 months, ocular herpes within 3 months, active ocular infection or inflammation, history of recurrent ocular inflammation within 3 months, eyelid abnormalities affecting function, ocular surface abnormalities compromising corneal integrity [46]	Adult [44]
**Intense pulsed** **light** (OptiLight)	Treatment of conditions such as MGD, rosacea, and telangiectasia, which may lead to DED [47,48]	Yes [49]	Every 4 weeks as needed [48]	Photothermolysis: uses specific wavelengths from pulsed light source to destroy unwanted blood vessels by targeting their chromophores [47]	Acute ocular inflammation [50]	No studies evaluating efficacy or safety in children for treatment of MGD [51]
**Low-level light therapy** (Epi-C Plus^®^)	Treatment of MGD contributing to DED [52]	Yes [38]	Twice weekly for at least 1 month [53]	Photobiomodulation: irradiation with nonionizing light to stimulate meibomian gland ATP production leading to endogenous healing and promoting meibum flow [52]	Eyelid margin tattoos [52]	No randomized clinical trials for treatment of MGD or DED [52]
**Microblepharo-exfoliation** (BlephEx^®^, AB Max™)	Treatment of *Demodex* infestation, blepharitis, and MGD leading to DED [54–56]	Yes	Every 4–6 months as needed [57]	Microsponge cleaning of the lid margin and lashes for removal of biofilm, bacteria, *Demodex*, and toxins promoting inflammation [55]	None [58]	Safe for use in pediatric patients^a^
**Device-based therapies**						
**Moisture chamber goggles**	Treatment of MGD and DED [59,60]	No [61,62]	Twice daily for at least 3 weeks [62]	Steam transfers heat to soften meibomian gland secretions, and moisture helps retain tear fluid [2,62]	None^a^	No clinical studies evaluating efficacy or safety in children
**Warm compresses**	Treatment of obstructive MGD associated with DED [63]	Yes [64]	Twice daily for at least 2 weeks [59,63]	Heat softens or liquefies secretions obstructing meibomian glands [2]	Use caution in patients with keratoconus or corneal thinning [65]	Safe for pediatric use [43]
**Eyelid hygiene devices** (NuLids™)	Treatment of MGD, blepharitis, and DED [66]	No	Once daily for at least 30 days [66]	Cleaning tip oscillates to facilitate removal of bacteria, mites, and dry skin from eyelid margin and opening of meibomian gland orifices [66]	None [66]	No clinical studies evaluating efficacy or safety in children [66]
**Neurostimulation device** (iTear^®^100)	Increase acute tear production [67]	Yes [67,68]	30 s twice per day for up to 30 days [67,69]	Vibratory stimulation of external nasal nerve increases tear production by activating nasolacrimal reflex [67,68]	None [70]	Adult [67]

^a^Based on the authors’ clinical experiences.

ATP: adenosine triphosphate; DED: dry eye disease; FDA: US Food and Drug Administration; MGD: meibomian gland dysfunction.

Many potential environmental and lifestyle modifications may be implemented to improve DED [2]. Environmental adjustments include increasing indoor humidity levels and avoiding desiccating conditions that may exacerbate DED, such as air conditioning, open car windows, extended airline flights, or blowing fans [2,3]. Additionally, individuals frequently using computer stations should implement ergonomic adjustments such as lowering the screen, which allows a downward gaze that decreases exposure of the ocular surface to the air and reduces tear film evaporation [2]. Lifestyle modifications include implementing daily eyelid and eyelash cleansing routines to decrease irritant and allergen exposure and reduce bacterial buildup, taking frequent screen breaks to ensure adequate blinking for cornea-protecting tear film distribution, and using appropriate prescription eyewear to prevent eye strain [2,71]. Maximizing general wellness by ensuring adequate sleep, hydration, nutrition, exercise, and psychological wellbeing may also help to improve the DED disease state, reduce any associated stress, and mitigate symptoms [2]. Environmental and lifestyle adjustments are often among the first recommended DED treatment interventions; compliant adherence to these principles is vital for long-term success [2,71].

### The role of primary care clinicians in dry eye disease management

As is the case with many medical conditions, PCCs are often the first points of contact for and evaluators of patients with DED. In this role, PCCs have the opportunity to provide essential preliminary patient education about the underlying disease process [2,15]. Effective patient education facilitating realistic expectations and encouraging long-term treatment adherence is essential for successful DED management [2,16,71]. Additionally, PCCs may be the first to suspect or diagnose DED and may initiate nonspecialized treatment or provide recommendations to help mitigate symptoms and minimize ocular surface damage [15,16]. For example, PCCs may treat underlying disorders thought to be contributing to DED, or may consider medication discontinuation, dose adjustments, or alternative options for agents that increase the risk of DED [2,15]. They might recommend conservative interventions such as ocular lubricants or warm eyelid compresses, or suggest individualized environmental modifications and/or lifestyle adjustments such as dietary changes that improve DED [15]. After initial diagnosis and patient education, PCCs should provide patient referrals to an ophthalmologist and/or optometrist as soon as possible for more in-depth evaluation and initiation of any necessary specialized treatment; thereafter, these eye care specialists most often will continue to manage the condition long term [15,16].

### Considerations for primary care clinicians in the management of dry eye disease

As previously discussed, a multitude of factors contribute to the development of DED, including ocular and systemic diseases, topical and systemic medications, and environmental influences [2,3,5]. Thus, it is important that practitioners carefully consider patient histories and implement individualized treatment protocols for patients affected by or at risk for DED to avoid exacerbating the condition as much as possible. For example, when selecting medications for treatment of comorbidities in patients with DED, PCCs may consider using agents with fewer ocular surface effects, implementing dose modifications, or prescribing less drying topical rather than systemic products when available [2].

Topical ocular medications may promote the development of DED due to their potential allergic, toxic, or inflammatory effects that disrupt the tear film layer, inhibit aqueous secretion by the lacrimal glands, or damage the ocular surface epithelium, corneal nerves, or eyelids [72]. For example, glaucoma medications or other topicals containing preservatives like benzalkonium chloride may contribute to DED by inducing ocular surface irritation and damage leading to dysfunction [2,72–74]. In high risk patients requiring these medications, PCCs may consider alternatives like preservative-free agents or agents with less harmful preservatives (e.g. sodium chlorite, sodium perborate, or polyquaternium-1) [2,75]. Other topical medications potentially promoting DED include prostaglandin analogues, which are used in the treatment of glaucoma or eyelash hypotrichosis and may cause obstructive meibomian gland dysfunction leading to DED [76–78]. Topical medications with an osmolarity or pH that disturbs the normal tear film may also worsen DED [72].

A large number of commonly prescribed systemic medications contribute to the development of DED; these may do so by inhibiting tear production, affecting sensory nerve signals and reflex tear secretion, triggering secretory gland inflammation, or irritating the ocular surface through secretion in tears [72]. Such medications include, but are not limited to, antidepressants, antipsychotics, anxiolytics (such as benzodiazepines), oral contraceptives, hormone replacement agents, analgesics/antipyretics, nonsteroidal anti-inflammatory drugs (NSAIDs), systemic and inhaled corticosteroids, antihistamines, diuretics, and isotretinoin [2,72,79]. Gomes et al. (2017) provides complete lists of topical and systemic medications contributing to DED [72].

### Considerations for clinical specialists in the management of dry eye disease

Consideration of the risk of DED is important in a wide variety of specialties due to the large number of contributory comorbidities and concomitant medications. Practicing clinicians should be familiar with DED and how it interrelates with their field. Key considerations for certain specialties are described below.

#### Psychiatrists

Many psychoactive medications, including antidepressants, antipsychotics, and anxiolytics, predispose patients to DED [72,80,81]. The mechanisms responsible for this are not definitively established and likely vary by drug class [82,83]. For example, tricyclic antidepressants likely cause DED by binding muscarinic acetylcholine receptors in the lacrimal gland and conjunctival goblet cells, decreasing their secretory function [83,84]. Selective serotonin reuptake inhibitors (SSRIs) have minimal anticholinergic activity but may promote DED by increasing the amount of serotonin and inflammatory mediators in the tears, causing dysfunction of and damage to ocular surface structures [83,85,86]. Serotonin and norepinephrine reuptake inhibitors (SNRIs) such as venlafaxine and duloxetine may be less drying antidepressant options due to their noradrenergic action; in a study by Koçer et al. (2015), patients taking SSRIs had greater reductions in tear production and were more likely to have DED than patients taking SNRIs [82,83].

Psychoactive medications can promote DED, and patients with DED have increased incidences of stress, anxiety, depression, and sleep and mood disorders, which, in turn, may require treatment [2,8,87,88]. Thus, appropriately managing DED may be integral to optimizing mental health.

#### Neurologists

Similarly to tricyclic antidepressants, many antiparkinsonian medications, such as levodopa, pramipexole, and benztropine, have anticholinergic activity leading to abnormal tear production and DED [72,84]. Additionally, numerous neurological disorders, including but not limited to stroke, paralysis, migraines, myasthenia gravis, diabetic neuropathy, and neurotrophic keratitis, are associated with tear film abnormalities and DED due to potential impairment of sensory and/or motor ocular innervation [89–92]. However, patients affected by DED are not always symptomatic for this condition [93]. Therefore, in addition to addressing the underlying disease process, neurologists should consider referring patients to an eye care specialist for periodic ocular surface evaluation [93].

#### Rheumatologists

Autoimmune disorders such as Sjögren syndrome, rheumatoid arthritis, and systemic lupus erythematosus are known risk factors for DED [3,89,94]. These conditions are thought to contribute to DED by causing immune cell infiltration and increased inflammatory cytokine expression in ocular structures including the lacrimal glands, conjunctiva, and cornea, which lead to inflammation, tissue dysfunction or destruction, and failure of normal tear production [3,95–97]. Regular ocular surface evaluation should be considered for these patients [95]. Medications used to treat rheumatic conditions, such as NSAIDs and corticosteroids, are also associated with increased incidence of DED [72,80,81,98,99]. Aspirin and ibuprofen are secreted in the tears and may induce tear film abnormalities in this way; NSAIDs may also be associated with decreased corneal sensitivity, which can impair normal tear production [100,101].

#### Dermatologists

Chronic facial and ocular rosacea are commonly associated with ocular disorders including blepharitis and meibomitis, which may lead to meibomian gland dysfunction and subsequent atrophy [102,103]. This causes tear film instability and decreased tear production [102]. Additionally, patients with ocular rosacea have greater concentrations of proinflammatory cytokines on the ocular surface, which commonly results in DED [102]. When rosacea-induced DED is severe or accompanied by corneal ulcers and/or scarring, affected patients may experience permanent visual impairment [102].

Common dermatologic medications may also predispose patients to DED. For example, isotretinoin (13-*cis*-retinoic acid), which is used topically and/or systemically for the treatment of aging and acne vulgaris, can cause blepharitis, meibomian gland dysfunction, and DED [72,104,105]. Isotretinoin is secreted by the lacrimal gland into the tears; in the sebaceous meibomian glands, it then isomerizes into *all-trans* retinoic acid, which causes meibomian gland dysfunction and atrophy [72,104,105]. This leads to reduced quality of meibomian gland secretion, reduced tear film stability (increased tear osmolarity and decreased TBUT), and worsened symptoms of DED [72,104]. These isotretinoin-induced changes to the meibomian glands are reversible with cessation of treatment [105,106]. Interestingly, isotretinoin does not appear to affect lacrimal gland secretion, signifying that DED induced by isotretinoin is likely due to meibomian gland deficiency rather than aqueous tear deficiency [72,105,107]. Isotretinoin may also be harmful to the conjunctiva and cornea. Patients receiving isotretinoin were found to have decreased density of conjunctival goblet cells with reduced intercellular contact and increased nucleus:cytoplasm ratio; this may alter mucin formation and thereby disrupt the lipid layer of the tear film [105,108,109]. Additionally, patients receiving isotretinoin were shown to have statistically significant decreases in corneal sensitivity after 3 months of treatment [110]. This can impair protective ocular mechanisms including blinking and tearing responses, and potentially lead to ocular surface damage [105].

#### Obstetrician-gynecologists

The association between hormone therapy and increased incidence of DED is seen in multiple studies [72,79,80,111]. Schaumberg et al. (2001) noted that hormone replacement therapy (HRT), especially with estrogen alone, conveyed an increased risk of DED in postmenopausal women and that the risk of DED increased with duration of HRT [111]. Chia et al. (2003) also found that HRT was associated with increased risk of DED in Australian individuals aged ≥49 years [80]. Hormonal contraceptive use in women also contributes to DED; increased risk is noted with regular use and higher quantities of contraceptives used [79]. The underlying mechanism responsible for the association between hormone therapy and DED is not yet known [79]. Possible explanations are that drug binding to meibomian gland estrogen receptors prevents normal secretion and/or that progesterone alters molecular processes essential to lacrimal and meibomian gland function [79]. Alternatively, decreased testosterone levels, which are often found in peri- and postmenopausal women and women using hormonal contraceptives, may promote the development of DED, since androgen binding to meibomian gland sex steroid receptors stimulates lipid synthesis and glandular secretion [112–115].

#### Pediatricians

The prevalence of DED in pediatric patients is likely underestimated due to the shortage of epidemiological data for this population, the challenges of symptom interpretation in younger individuals, and the tendency of practitioners to associate DED only with congenital or autoimmune disorders in these patients [43]. Pediatric DED prevalence estimates vary widely depending on the examined population and presence of preexisting risk factors; values range from 0.2% in patients aged 2–17 years in the general US population to 97.5% in patients aged 3–6 years with preexisting allergic conjunctivitis in China [43,116,117]. Gupta et al. (2018) found that 42% of predominantly white US children aged 4 to 17 years who were asymptomatic and without a previous diagnosis of DED had meibographic evidence of glandular atrophy [118]. Risk factors promoting DED in pediatric patients include congenital disorders like congenital corneal anesthesia and cystic fibrosis; autoimmune diseases such as Sjögren syndrome and juvenile rheumatoid arthritis; inflammatory disorders like ocular allergy; dermatologic conditions such as ocular rosacea and ocular demodicosis; nutritional deficiencies of vitamin A, zinc, vitamin D, or omega fatty acids; and environmental or lifestyle factors like contact lens wear [19,43,116,119–127]. Kocamiş et al. (2021) and Moon et al. (2016) found a correlation between increased screen time and risk of developing DED in pediatric patients, which is significant considering the increasing dependence on digital devices in this population [128,129].

Diagnosing DED is challenging in the pediatric population due to the difficulties of identifying symptoms, the lack of validated diagnostic criteria and normative data, and absence of tests specifically designed for pediatric patients [19,43]. Typically, the presence of corresponding symptoms plus at least 1 objective marker of DED (decreased TBUT, increased tear osmolarity, or positive ocular surface staining) are the key findings used to establish a diagnosis of pediatric DED [43]. The mainstays of initial treatment are similar to those of adults and include patient and/or caregiver education, environmental and/or lifestyle modifications, nutritional support, eyelid hygiene and/or warm compresses, and use of ocular lubricants [19,43]. Any underlying conditions contributing to DED should also be addressed [19]. Prescription medications and/or procedural interventions may be implemented in moderate to severe cases with failure of these first-line therapies [19,43].

Due to the many challenges of diagnosing and managing DED in pediatric patients, referral to an eye care specialist is recommended as early in the course of disease as possible in suspected cases [19,124]. Delays in diagnosis and treatment can lead to potentially serious complications, including corneal surface irregularities, epithelial defects, neovascularization, infectious ulcers, and corneal scarring, all of which may cause vision loss in these young patients [120].

## Conclusions

Primary care clinicians, as well as many clinical specialists, play a vital role in DED management by educating patients about the disease process, establishing a diagnosis and potentially prescribing nonspecialized treatment, and providing referrals to eye care specialists for evaluation and initiation of chronic therapy. Successful DED management often involves the use of multiple pharmacologic and/or nonpharmacologic therapies, as well as environmental and lifestyle adjustments, to address any underlying etiologies and restore and preserve tear film homeostasis in affected patients. Primary care clinicians and clinical specialists should carefully consider their treatment protocols for patients at risk of or with existing DED and, when possible, utilize medications with fewer ocular surface effects to minimize ocular surface damage.

## Data Availability

Data sharing is not applicable to this article because it is a narrative review and as such, no new data were created or analyzed in this study.
